# Crystal structure of human INPP5K with an allosteric inhibitor reveals the structural basis for species specific potency

**DOI:** 10.1038/s41598-026-40748-4

**Published:** 2026-02-26

**Authors:** Akihiro Nomura, Keishi Yamaguchi, Motoaki Kawano, Kazuki Hanada, Jun Nishihata, Masato Noguchi, Tsuyoshi Adachi

**Affiliations:** 1 Chemical Research Laboratories, Central Pharmaceutical Research Institute, Japan Tobacco Inc., 1-1, Murasaki-Cho, Takatsuki Osaka, 569-1125 Japan; 2 Biological Pharmacological Research Laboratories, Central Pharmaceutical Research Institute, Japan Tobacco Inc., 1-1, Murasaki-Cho, Takatsuki Osaka, 569-1125 Japan; 3 Pharmaceutical Frontier Research Laboratories, Central Pharmaceutical Research Institute, Japan Tobacco Inc., 1-13-2, Fukuura, Kanazawa-Ku, Yokohama Kanagawa, 236-0004 Japan

**Keywords:** Inositol polyphosphate 5-phosphatase K (INPP5K), Allosteric inhibitor, Crystal structure, Drug development, Species-specificity, Phosphatase, Structural biology, Biochemistry, Physiology, Structural biology

## Abstract

Inositol polyphosphate 5-phosphatase K (INPP5K) is a phosphatidylinositol (3,4,5)-trisphosphate phosphatase that increases glucose uptake and regulates myogenesis in the skeletal muscle. To understand the mechanism of its species-specific inhibition, we determined the 1.9-Å resolution crystal structure of human INPP5K in complex with a selective inhibitor, CPD-1 (IC_50_ = 2.9 µM). The structure reveals that CPD-1 binds to a novel allosteric pocket, inducing a large conformational change in α-helix 3 that alters the active site and prevents substrate binding. This finding explains its unique, noncompetitive inhibitory mechanism. Crucially, while the inhibitor-binding residues are conserved, the key residue governing the allosteric transition is not conserved in mouse and rat INPP5K, which correlates with their insensitivity to CPD-1 (IC_50_ > 100 µM). Based on these structural insights, we identified the hamster as a pharmacologically relevant preclinical model (IC_50_ = 8.2 µM). These findings provide a structural basis for the rational design of next-generation INPP5K inhibitors and establish a suitable animal model for their evaluation.

**Supplementary Information:**

The online version contains supplementary material available at 10.1038/s41598-026-40748-4.

## Introduction

Phosphatidylinositol (3,4,5)-trisphosphate (PtdIns(3,4,5)P₃) recruits and regulates the activation of effector signal transduction proteins containing pleckstrin homology (PH) domains^1^. These effector proteins regulate a number of cell functions, including cell migration, tissue growth, and glucose metabolism^2^.

Inositol polyphosphate 5-phosphatase K (INPP5K), formerly known as skeletal muscle- and kidney-enriched inositol polyphosphate 5-phosphatase (SKIP), is a type II phosphatase (inositol polyphosphate 5-phosphatase (IP5Pase)) that hydrolyzes the P-5 position of PtdIns(4,5)P_2_ and PtdIns(3,4,5)P_3_^3^. The INPP5K substrate PtdIns(3,4,5)P_3_ is generated by class IA phosphatidylinositol 3-kinase (PI3K) in response to stimulation by growth factors and insulin. INPP5K is expressed abundantly in skeletal muscle and also in the brain, heart, and kidney, and possesses an N-terminal IP5Pase catalytic domain and a C-terminal SKICH (SKIP C-terminal homology) domain. Its localization to the endoplasmic reticulum is mediated by this SKICH domain under basal conditions, but it translocates to plasma membrane ruffles in response to insulin, where it negatively regulates insulin-dependent glucose uptake into skeletal muscle by downregulating PI3K/AKT signaling^4,5^. Heterozygous INPP5K knockout mice have been reported to be highly insulin-sensitive, to show an enhancement of insulin signaling in skeletal muscle, and to have larger amounts of muscle than wild-type mice^6^. Furthermore, knockdown studies have shown that INPP5K negatively regulates myogenesis through the inhibition of insulin-like growth factor-II (IGF-II) production and attenuation of the IGF-II/AKT/mechanistic target of rapamycin signaling pathway^7^. INPP5K is also thought to be a key regulator of muscle cell differentiation, and recently, it has been reported that mutations in INPP5K cause congenital muscular dystrophies (CMDs)^8^.

A typical IP5Pase has several loops and helices in the outer shell of its core, composed of β-sheets. IP5Pases such as inositol polyphosphate 5-phosphatase E (INPP5E)^1,2,4^, oculocerebrorenal syndrome of Lowe protein (OCRL)^5–7^, and proline-rich inositol polyphosphate 5-phosphatase (PIPP) are characterized by the presence of a highly conserved substrate-binding site at one terminus of the β-sheet bundle in the center core region, and substrate interactions involve the loops and short helices connecting each β-sheet^9–11^. While several allosteric inhibitors of phosphatases have been identified, their structural mechanisms often remain unclear. Here, we report the crystal structure of human INPP5K in complex with a synthetic, species-specific inhibitor. Our analysis reveals how the binding of this compound directly induces a dislocation of α-helix 3, uncovering a novel allosteric mechanism of inhibition. By comparing this structure with sequences from different species, we clarify the molecular basis for the inhibitor’s species specificity. These findings not only provide a blueprint for structure-based drug design but also establish a rational basis for selecting the most appropriate animal model for preclinical studies.

## Results

### Alignment of the amino acid sequences of members of the human IP5Pase family

**Fig. 1:**
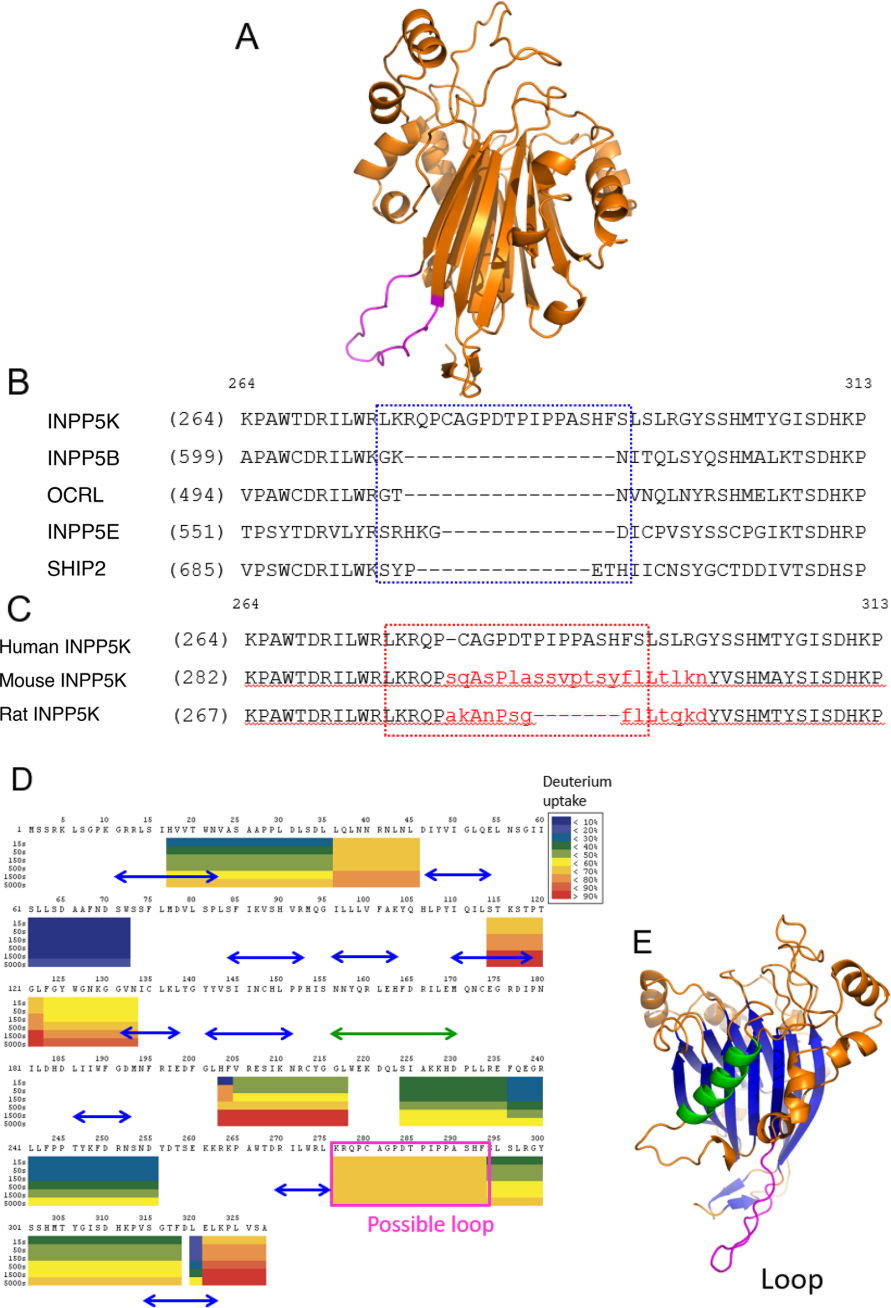
Sequence analysis and flexibility mapping of the INPP5K catalytic domain. (**A**) Structural model of human INPP5K-cd, generated using Boltz-2^12^, highlighting the predicted flexible loop region (magenta) corresponding to the sequence indicated in (**B**). (**B**) Sequence alignment showing the insertion region unique to INPP5K among human IP5Pases. (**C**) Sequence variations in the predicted loop region among human, mouse, and rat INPP5K. Residues differing from the human sequence are shown in red. The region targeted for substitution is indicated by the red dotted line. (**D**) Heat maps display the mean % deuteration of fragments of human INPP5K-cd. Each color block represents an analyzed peptide fragment. The deuteration levels of peptides are differently colored as blocks in the insert at six time points (15, 50, 150, 500, 1 500 and 5 000 s). The colors of the double-headed arrows correspond to those used in the structure model in E. The green arrow and blue arrows indicate the alpha-helix and beta-sheets, respectively. The predicted loop fragment is shown in a square magenta box. The predicted flexible loop fragment (residues 276–293) is boxed in magenta. (**E**) Ribbon diagram of the INPP5K-cd structural model, colored according to the results of the HDX–MS analysis. The central beta-sheet core is shown in blue, the major alpha-helix in green, and the predicted flexible loop (residues 276–293) in magenta. Other structural elements, including the connecting loops and smaller helices, are shown in orange.

The sequence identities of the INPP5K IP5Pase domain and other IP5Pase domains are as follows: inositol polyphosphate 5-phosphatase B (INPP5B), 35%; OCRL 32%; SH2-containing inositol 5’-phosphatase 2 (SHIP2), 27%; and INPP5E, 25%^9–11^. We identified an extra sequence in the middle of INPP5K by comparing these IP5Pases with respect to their protein sequences (Supplementary Fig. 1). This extra region, which is not present in other IP5Pases, may indicate the presence of a wobble loop. When a model of the INPP5K catalytic domain (cd) was studied using Boltz-2^12^, we identified a large flexible loop region corresponding to this extra sequence in INPP5K (Fig. 1A–C). Furthermore, Hydrogen/Deuterium-exchange (HDX)–mass spectrometry (MS) analyses showed that the region would be flexible, indicated by rapid deuterium uptake at any incubation time in D₂O (Fig. 1D and E, Supplementary Fig. 2).

The sequence identities of the INPP5K IP5Pase domain between hfiumans and other species were as follows: rat INPP5K, 75% and mouse INPP5K, 76%. The INPP5K sequence identity between mouse and rat is 89% (Supplementary Table 1). The notable differences between these species were found to be present in the predicted loop region of the IP5Pase domain. When the purified IP5Pase domains were evaluated by SDS-PAGE, rat INPP5K generated a sharp, well-defined band at its expected molecular weight, whereas the mouse protein produced a broader, more diffuse band. This observation suggests that the rat protein was more conformationally homogeneous than its mouse counterpart, making it more favorable for crystallization (Supplementary Fig. 3). This implies that the loop region in rat INPP5K is less flexible than that in mouse INPP5K.

### Results of the HDX–MS study of human INPP5K-cd

To further characterize the flexible region of human INPP5K-cd, we evaluated deuterium uptake by this region. The percentage deuterium uptake was calculated at six time points for each of the peptides produced by pepsin digestion and visualized in the sequence coverage map for the human INPP5K-cd (Fig. 1D, Supplementary Fig. 2). The HDX kinetics for peptides from the same region with different lengths and charge states were included. The sequence coverage was 45%. To characterize the flexible loops effectively, we used mild protease-digestion conditions, and the lower coverage rate can be attributed to incomplete proteolytic digestion in the absence of detergent. As predicted, the β-sheet regions and some rigid α-helices were not digested. Overall, the peptides detected on MS analysis showed a moderate level of HDX. The amino acid sequences 36–45, 114–133, 276–293, and 321–328 showed prompt HDX from very early time points. Of these, the 276–293 region was not conserved in the other IP5Pases or in other species (Supplementary Figs. 1 and 3). On this basis, we concluded that the 276–293 region is a highly flexible part of the tertiary structure of human INPP5K-cd. Therefore, part of this region was replaced with the relatively short sequence from rat INPP5K-cd, and the resulting mutant human INPP5K-cd (Δ INPP5K cd, 1–322, Δ285–290) was subjected to crystallization (Fig. 1C).

### Inhibitory effects of the deletion mutant h INPP5Ks

**Fig. 2:**
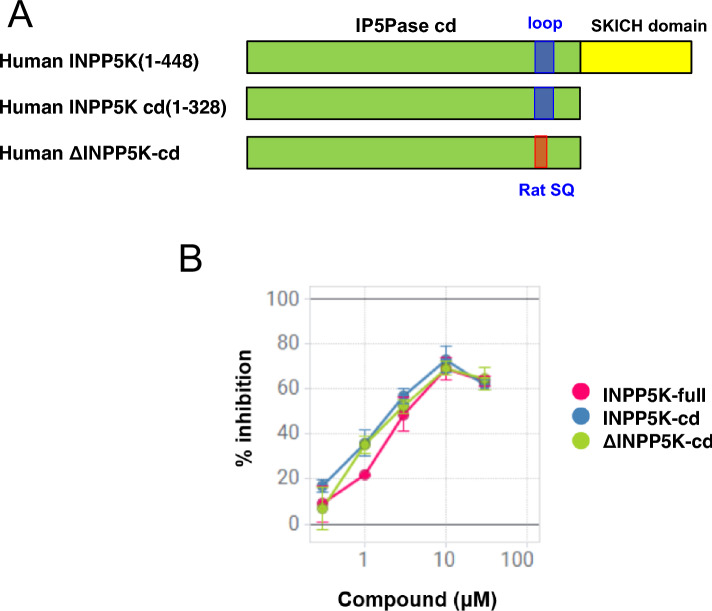
Domain organization of the INPP5K constructs and the inhibitory effects of CPD-1. (**A**) Schematic domain structures of full-length human INPP5K (INPP5K-full), the catalytic domain (INPP5K-cd), and the loop-substituted catalytic domain (delta-INPP5K-cd) used for crystallization. The IP5Pase catalytic domain (green), SKICH domain (yellow), predicted flexible loop region (blue), and region substituted with the rat sequence (red) are indicated. (**B**) Dose–response curves for the inhibition of INPP5K-full (pink curve, IC50 = 3.7 µM), INPP5K-cd (blue curve, IC50 = 2.3 µM), and delta-INPP5K-cd (light green curve, IC50 = 2.9 µM) by CPD-1, determined using a fluorescence polarization assay. Data are presented as mean ± SD from three independent experiments (n = 4).

CPD-1 (IC₅₀ = 2.9 µM) was identified as a specific inhibitor of INPP5K by high-throughput screening (HTS)^3^ and then evaluated using an inorganic phosphate detection assay, as shown in Table 1. The compound displayed noncompetitive inhibition of human INPP5K, but no inhibition of mouse or rat INPP5K. First, we determined whether or not the human INPP5K-cd and Δ INPP5K-cd displayed comparable 5-phosphatase activities and were inhibited to the same extent (Fig. 2, see Methods). To confirm that the deletion in the flexible loop did not alter the protein’s sensitivity to the inhibitor, we compared the inhibitory effects of CPD-1 on the wild-type INPP5K-cd and the Δ INPP5K-cd mutant. Both proteins displayed nearly identical dose–response curves (Fig. 2B). This result indicates that the deletion in the loop region does not affect the compound’s inhibitory mechanism. Therefore, we concluded that the Δ INPP5K-cd construct was a valid surrogate for the wild-type protein for structural studies and proceeded with its crystallization.

**Table 1:** IC_50_s for CPD-1, identified to be a highly selective inhibitor of human INPP5K.

CPD-1	Human INPP5K	Hamster INPP5K	Guinea pig INPP5K	Mouse INPP5K	Rat INPP5K	Human SHIP2	Human PIPP
	2.9	8.2	59.0	> 100	> 100	> 100	> 100

### Overall structure of the deletion-mutant INPP5K-cd in complex with CPD-1

**Fig. 3:**
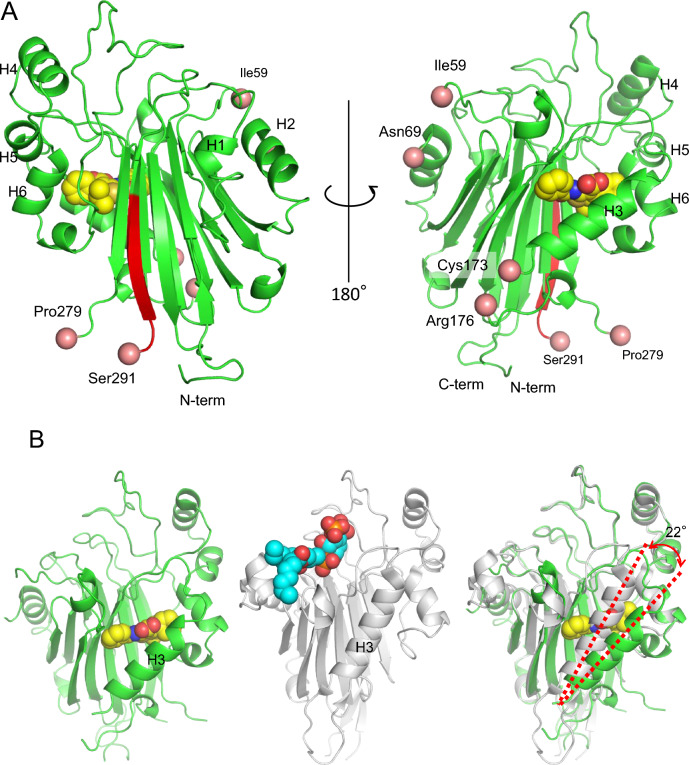
Overall structure of human delta-INPP5K-cd complexed with CPD-1. (**A**) Cartoon representation of human delta-INPP5K-cd, with helices numbered from the N- to the C-terminus (left: front view, right: rotated 180 degrees vertically). The region substituted with the rat sequence is shown in red. CPD-1 is shown as spheres (carbon: yellow, nitrogen: blue, oxygen: red, sulfur: brown). The N- and C-termini of the modeled regions are indicated by pink spheres. (**B**) Structural comparison with INPP5B. (Left) Structure of the delta-INPP5K-cd/CPD-1 complex (green). (Middle) Structure of INPP5B bound to PtdIns-3,4-P2 (PDB: 4CML, gray). (Right) Superimposition of the delta-INPP5K-cd/CPD-1 complex onto the INPP5B complex. The ligand PtdIns-3,4-P2 is shown as spheres (carbon: pale blue, oxygen: red, phosphorus: orange). H3 in the INPP5K complex structure is displaced outwards by inhibitor binding.

We next investigated the noncompetitive inhibitory mechanism of the compound by determining the 1.9-Å resolution structure of the human Δ INPP5K-cd (1–322, Δ285-290)/CPD-1 complex. The data and the refined structures are summarized in Table 2. Although repeated efforts to obtain the structures of complexes of wild-type INPP5K-cd (1–328) with several compounds were unsuccessful, we successfully grew crystals of the complex between the inhibitor of interest and the Δ INPP5K-cd construct. The overall structure was quite similar to that of INPP5B. Several α-helices and loops comprised the outer shell surrounding the core, which was comprised of β-sheets (Fig. 3A). The binding of the inhibitor between the largest helix and β-sheets induced outward tilting of the α-helix at a hinge at the C-terminal end of the helix (Fig. 3B).

**Table 2:** Crystal characteristic, data collection, and refinement statistics for human Δ INPP5K-cd.

CPD-1	
Space group	*P3*_*2*_*21*
**Unit-cell parameters**	
a, b, c (Å)	82.28 82.28 107.31
α, β, γ (°)	90.00 90.00 120.00
Resolution range (Å)	71.25 – 1.90 (2.26 – 2.20)
Total reflections	31 892
Unique reflections	30 947
Completeness (%)	100.0 (100.0)
Redundancy	21.3 (5.7)
I/δ (%l)	7.5 (1.8)
R_merge_ (%)	0.098 (0.435)
**Refinement statistics**	
Resolution range (Å)	2.20 – 101.28
No. of reflections	29 357
R_cryst_ (R_free_)	16.86 (22.22)
**No. of atoms**	
Protein	3 810
Ligand	66
Water	321
**B-factors**	
Protein	27.972
Ligand or Ion	16.938
Water	34.320
**R.m.s. deviations**	
Bond length (Å)	0.018
Bond angles (˚)	1.771

Values in parenthesis are for the highest-resolution shell.

^b^Rmerge = ∑|*I*_h_-(*I*)_h_|/∑*I*_h_, where (*I*)_h_ is mean intensity over symmetric equivalents.

^c^R-factor = ∑|*F*_obs_-*F*_calc_|/|∑|*F*_obs_. The free R-factor was calculated from 10% of the reflections that were omitted from the refinement.

**Fig. 4:**
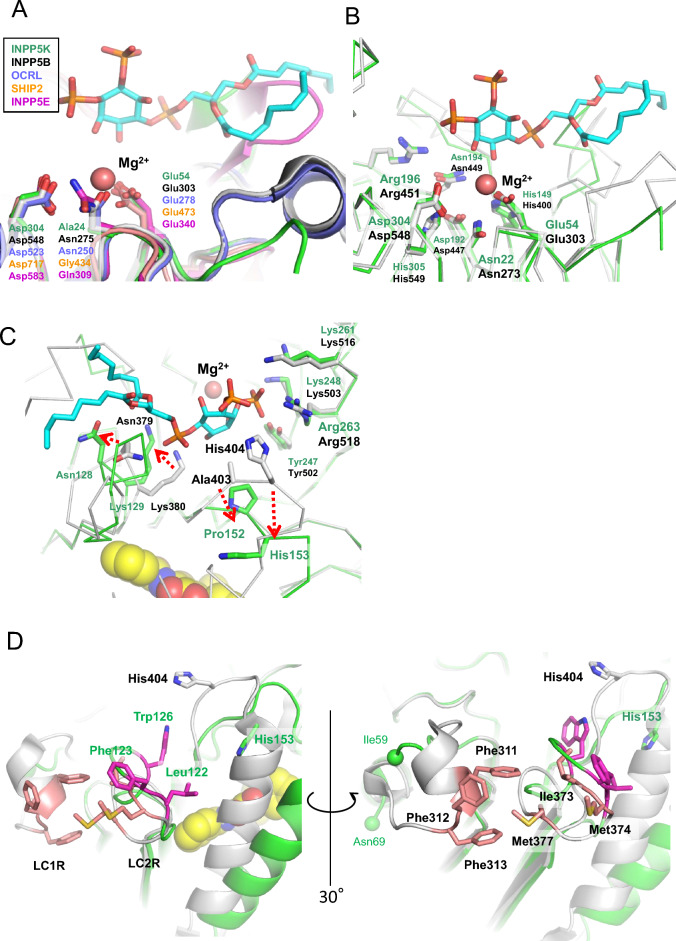
Comparison of the active site and potential membrane interaction regions. (**A**) Comparison of the predicted Mg2 + -binding site in human delta-INPP5K-cd (green) with INPP5B (PDB: 4CML, gray), OCRL (PDB: 4CMN, purple), SHIP2 (PDB: 5OKM, orange), and INPP5E (PDB: 2XSW, magenta). Key residues are shown as sticks. (**B**) Comparison of the catalytic site residues between delta-INPP5K-cd (green) and INPP5B (gray). (**C**) Both the inositol-ring interaction loop involving Pro152 and His153 and the 1-phospate interaction loop involving Asn128 and Lys129 were found to be substantially shifted. Superimposition onto INPP5B indicated that the two loops are displaced from their original position, as shown for INPP5B (red dotted arrows). (**D**) Comparison of potential lipid interaction motifs (LC1R and LC2R). View rotated approximately 30 degrees vertically. The amino acid residues of LC1R and LC2R in INPP5B are shown in pink, and the amino acid residues of LC2R in INPP5K are shown in magenta. The amino acid residues of LC1R are invisible between Ile59 and Asn69. PtdIns-3,4-P2 is depicted as stick model with carbon atoms in pale blue, phosphorus atoms in orange, and oxygen atoms in red.

The obtained crystal belonged to the *P3₂21* space group. In the refined structure, no interpretable electron density was observed for the loop region spanning residues Ala279 to Pro284, and thus these residues could not be modeled. This lack of ordered structure is consistent with our earlier HDX-MS analysis, confirming the high degree of flexibility in this region (Fig. 3A). Residues in the possible loops from Ile60 to Phe68 and two residues from Glu174 to Gly175 were also not visible. In the crystal packing, the region that was substituted using the mouse homolog was located in an open space and seemed not to be able to be in contact with a neighboring molecule. The predicted substrate-binding site in our structure remains accessible. A structural comparison shows that this site is well-aligned with that of the INPP5B-substrate complex, with the notable exception of the inositol ring recognition loop, which adopts a different conformation. Attempts to grow crystals of the apo protein (the enzyme alone) or in a complex with its enzymatic product, the dioctanoyl-phosphatidylinositol phosphate (diC8PtdInsP), were unsuccessful. This might be because we used crystallization conditions that involved malonate but no other substances. The malonate was located at the dioctanoyl-phosphatidylinositol 4,5-bisphosphate (diC8PtdInsP₂)- and Mg^2^⁺ ion-binding site (Fig. 4A and B, Supplementary Fig. 4). Malonate may sterically interfere with product-binding. Furthermore, the proline residue in the substrate-binding site of INPP5K may be also sterically hindered in the apo structure by malonate (Supplementary Fig. 4).

The catalytic mechanism of 5-phosphatase, which involves Mg^2^⁺ ions, has recently been proposed^10^. Mg^2^⁺ ions are located within 4 Å of the three charged amino acid residues in most human IP5Pases. The superimposition of five IP5Pase structures revealed that two of these residues are conserved, but Asn in INPP5B and OCRL replaced by Ala24 in INPP5K (Fig. 4A and B). However, the omit map obtained from the refined model showed no electron density that matched to the Mg^2^⁺ ion.

The amino acid residues in the probable substrate-binding site, with the exception of Pro152, were found to be well conserved. Both the inositol recognition loop, involving Pro152 and His153, and the 1-phosphate interaction loop, involving Asn128 and Lys129, were largely separate from the substrate-binding structure of INPP5B. These dislocations are likely to have been caused by inhibitor binding (Fig. 4C).

With respect to INPP5B and OCRL, the aliphatic region of the dioctanoyl-phosphatidylinositol phosphate (diC8PtdInsP) product primarily interacted with two hydrophobic loops: the lipid chain 1 recognition motif (LC1R motif) and the lipid chain 2 recognition motif (LC2R motif) (Fig. 4D). LC1R includes three consecutive Phe residues, whereas LC2R is composed of Ile, Met, and Met. The loop corresponding to the LC1R motif in INPP5K may have been in the missing region, and includes hydrophobic residues such as Leu or Ile. The loop corresponding to the LC2R motif in INPP5K dislocates following inhibitor binding, and may correspond to Leu122, Phe123, and Trp126 in INPP5K.

### Binding modes of the inhibitors

**Fig. 5:**
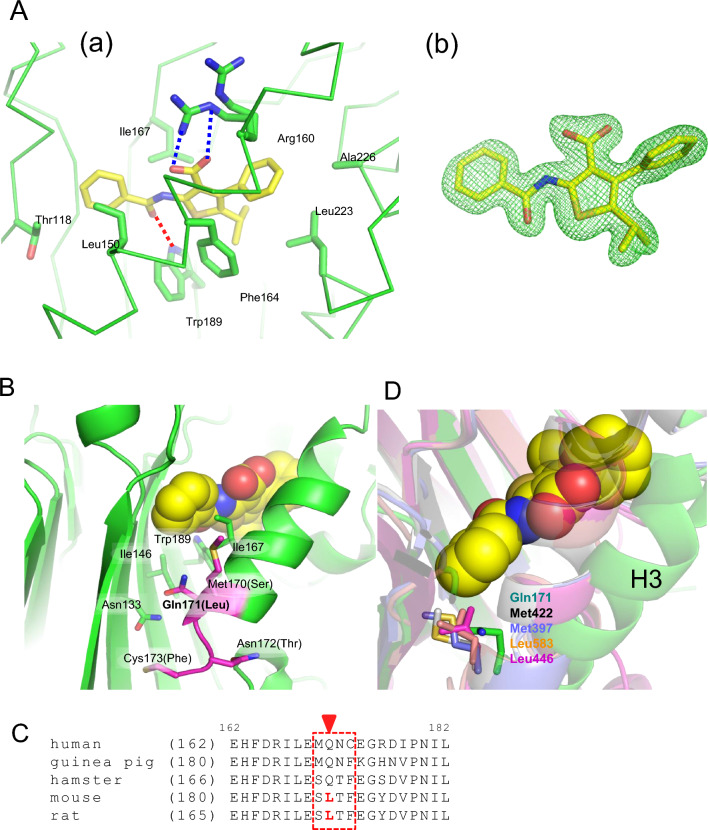
Binding mode of the allosteric inhibitor CPD-1. (**A**) Detailed view of the inhibitor-binding site in human delta-INPP5K-cd. CPD-1 (sticks, colored as in Fig. 3A) forms a hydrogen bond (dashed line) with Trp189 (backbone NH) and electrostatic interactions with alternative conformations of Arg160. Key interacting residues are shown as sticks. The mFo-DFc omit map for CPD-1 is shown as a green mesh, contoured at 2.0 sigma. (**B**) Surface representation of delta-INPP5K-cd, with residues in the hinge region of H3 that differ between human and mouse/rat INPP5K being highlighted. (**C**) Sequence alignment of the H3 regions of human, mouse, and rat INPP5K. The red arrowhead indicates Gln171 (human) which is replaced by Leu in mouse/rat INPP5K. (**D**) Structural comparison of the H3 C-terminal hinge regions of delta-INPP5K-cd (green), INPP5B (PDB: 4CML, gray), OCRL (PDB: 4CMN, purple), SHIP2 (PDB: 5OKM, orange), and INPP5E (PDB: 2XSW, magenta).

The crystallization of human Δ INPP5K-cd with CPD-1 yielded crystals only under a single condition. Unambiguous electron densities meant that the site of inhibitor binding between β-sheet bundles and H3 was clearly defined (Fig. 5A and B). The binding site of the inhibitor was quite different to that of the competitive inhibitors of other IP5Pases. Superimposition of INPP5B structure onto the INPP5K complex structure revealed that the helix was tilted approximately 22° outwards to accommodate ligands (Fig. 3B). This displacement led to a conformational change in the upstream loops, which are considered to be part of the substrate-binding site (Fig. 4C). Notably, a predicted model of the INPP5K/CPD-1 complex predicted by Boltz-2 failed to identify the allosteric site that was revealed by the crystal structure (Supplementary Fig. 5). Superimposition onto INPP5B complexed with diC8PtdInsP₂ (PDB code: 4CML) showed that conformational changes to the linking loops that are induced by inhibitors would affect substrate-binding affinity. These structural findings imply that the inhibitor binds to an allosteric site.

CPD-1 becomes associated with the hydrophobic region of the enzyme via an electrostatic interaction between its carboxylic group and the Arg160 side chain (Fig. 5A). This side chain has two alternative conformations in this binding mode. The hydrogen of the Trp189 side chain is bound to the oxygen atom in the amide group of CPD-1. In addition, the isopropyl group and phenyl group of the compound form hydrophobic interactions with Phe164, Ile167, Leu233, and Ala226, which line the binding pocket. Finally, the benzoyl amide group forms a hydrophobic interaction with Leu150 and Cβ of Thr118. Seven amino acid residues are positioned within 4 Å of CPD-1, facilitating close contacts.

### Structural characteristics of human INPP5K

CPD-1 (IC₅₀ = 2.9 µM) is a specific inhibitor of human SKIP, but not mouse or rat SKIP. We next aimed to characterize the structures of complexes with HTS hit compounds, but only the crystal for the complex with CPD-1 was obtained. Four amino acid residues in the inhibitor-binding pocket are commonly replaced by Leu in both the mouse and rat sequences (Fig. 5B and C). This region is located at the hinge of the α-helix movement. Gln171 is replaced by Leu in both mouse and rat, and by hydrophobic amino acid residues such as Leu or Met in INPP5B, OCRL, SHIP, and INPP5E (Fig. 5D). Gln171 at the C-terminus of the helix was thought to face the β-sheet core region, based on a comparison with INPP5B. We hypothesized that the replacement of Leu might have an important role in inhibitor binding.

We focused on two species that, like human SKIP, include Gln171. The inhibitor had comparable inhibitory effects on hamster SKIP (IC₅₀ = 8.2 µM), but showed weaker inhibitory effects on guinea pig SKIP (IC₅₀ = 59.0 µM). Sequence alignment of the binding site revealed that the amino acid residues directly interacting with CPD-1 are highly conserved across human, hamster, guinea pig, mouse, and rat (Supplementary Table 2). Although Thr118 shows variation (Thr in human and mouse vs. Ile in hamster and rat), this does not correlate with inhibitor sensitivity; for instance, the mouse enzyme is insensitive despite possessing the same Thr118 as the human enzyme, whereas the hamster enzyme is sensitive despite the T118I substitution. These results imply that Gln171 is primarily important for the tilting of H3 to accommodate the inhibitor. Therefore, the hamster may be a more useful animal model for the development of this allosteric inhibitor as a potential therapeutic substance than the mouse or rat.

## Discussion

We identified a novel, selective INPP5K inhibitor, CPD-1. This compound is a noncompetitive inhibitor of human INPP5K but is inactive against the mouse and rat orthologs. To investigate why the compound showed this species-specificity and to assist with future efficient structure-based drug design, we clarified the mode of action of this compound. The analysis of the crystal structure of complexes with the inhibitor provided insight into the most useful rodent species for the evaluation of the efficacy and pharmacokinetics of candidate drugs. We identified the hamster to be the most suitable species for compound evaluation, without evaluating the inhibitory activity of the candidate molecule with respect to recombinant proteins derived from many species by means of structural biology studies.

The studied inhibitor binds to the newly generated pocket after repositioning of H3. It is thought to tilt from its original position, which was extrapolated by the superimposition of the structure of INPP5B. This tilting motion of H3 would require a hinge in its terminus region, and in the present structural analysis, we identified a critical difference in this hinge region that could explain the species-specificity of the effects of the inhibitor: Gln171 in human INPP5K is replaced by a hydrophobic Leu residue in both the mouse and rat INPP5K (Fig. 5C).

In homologous proteins such as INPP5B, OCRL, SHIP, and INPP5E, the residue corresponding to position 171 is typically a hydrophobic Leu or Met (Fig. 5D). These residues are usually buried within the protein core, stabilizing the closed conformation through hydrophobic interactions and preventing solvent exposure; consequently, no allosteric sites have been reported for these enzymes. Similarly, in mouse and rat INPP5K, the hydrophobic Leu171 likely forms stabilizing hydrophobic interactions with residues such as Ile164 and Ile185, thereby “locking” the closed state. Therefore, the structural transition required for CPD-1 binding is energetically unfavorable due to the penalty associated with exposing the hydrophobic side chain of Leu171 to the solvent.

In contrast, while the polar Gln171 in human INPP5K may contribute to the closed state via van der Waals interactions or hydrogen bonds, its exposure upon helix H3 tilting allows for interaction with water molecules. This hydration capability likely significantly reduces the energetic penalty of the conformational change compared to Leu^13^. Thus, we propose that the allosteric pocket is formed exclusively in INPP5K possessing Gln171 because this residue facilitates the necessary structural transition.

This hypothesis is strongly supported by the comparison between hamster and mouse/rat orthologs. These species share high sequence similarity (approx. 87% identity, Supplementary Table 1). Crucially, a detailed comparison of the inhibitor-binding pocket reveals that key residues contacting CPD-1 are conserved between hamster and mouse/rat, with the non-critical exception of Thr118 and the critical exception of the gatekeeper residue at position 171 (Supplementary Table 2). This indicates that the species selectivity does not arise from direct differences in the binding interface but rather from the conformational energetics governed by the hinge region of helix 3 (H3). The key difference was found at the base of helix H3, which serves as the hinge for the conformational change. The fact that the hamster enzyme is sensitive (IC_50_ = 8.2 µM) while mouse/rat enzymes are insensitive (IC_50_ > 100 µM) serves as a "natural mutagenesis experiment," confirming that Leu171 is the primary cause of resistance. The reduced affinity in the guinea pig ortholog (IC_50_ = 59.0 µM), despite the presence of Gln171, provides further mechanistic insight. To elucidate this discrepancy, we analyzed the structure of guinea pig INPP5K using an AlphaFold 3 model. Structural comparison revealed that unique basic residues (Lys244 and Arg246) in the guinea pig loop region form specific salt bridges with aspartate residues (Asp176 and Asp183) on helix H3 (Supplementary Fig. 6). This interaction network likely acts as an "electrostatic brake," physically tethering helix H3 and potentially stabilizing the closed conformation. In contrast, comparative modeling of the hamster ortholog (IC_50_ = 8.2 µM) revealed that it shares key electrostatic features with the human enzyme. Specifically, the residue corresponding to the guinea pig’s braking Arg246 is replaced by an aspartate in both human (Asp230) and hamster (Asp234) enzymes. This substitution not only eliminates the tethering salt bridge but may also create electrostatic repulsion against the opposing acidic residues on helix H3 (Asp165 in human, Asp169 in hamster). This shared "repulsion-assisted" mechanism may destabilize the closed state, facilitating the conformational change. Thus, high-affinity inhibition likely requires both the flexible gatekeeper (Gln171) and a favorable electrostatic environment free from stabilizing constraints, as observed in both human and hamster enzymes.

This conclusion was further supported by the fact that most other IP5Pase family members contain a conserved hydrophobic residue (Leu or Met) at this position, which likely maintains a stable interaction with the β-sheet (Fig. 5D). Therefore, the unique, polar Gln171 is a key determinant of the specific allosteric inhibition of human INPP5K by the compound of interest.

The inhibitor of interest shows allosteric inhibitory effects on human INPP5K, and the identification of the location of binding of the inhibitor has improved our understanding of the mechanism of this noncompetitive inhibition. Superimposition of the INPP5B complex structure with PtdIns(3,4)P₂ revealed that the metal ions are closely superimposed, and suggested that the products are located in the same cleft as the conserved amino acid residues (Fig. 4C). The displacement of two loops occurs in the substrate-binding site: 1) the loop including Asn128 and Lys129 is the conserved 1-P moiety-interacting site, and 2) the Pro152 and His153 in the other loop are likely to be the sites of contact with the inositol-ring and the 3-P group, respectively. We speculate that the displacements induced by the inhibitor would cause a loss of contact with the substrate. The repositioning of H3 in the presence of the inhibitor creates a druggable allosteric site, and therefore offers a potential target for the development of selective inhibitors. This pocket is formed primarily through hydrophobic interactions with the compound. The carboxyl group of the compound forms an alternative electrostatic interaction with Arg160, which is exposed to the solvent. The alternative conformation of the Arg160 side chain may lead to a weaker interaction between Arg160 and carboxyl group, possibly because the N-atom of the amide may interfere with the interaction. Chemical compounds with greater bioactivity are being produced that have a strong electrostatic interaction with Arg160 through the study of structure–activity relationships.

The differences in the amino-acid composition of the substrate-binding site may determine the unique substrate specificity of INPP5K. In INPP5B and OCRL, Ala403 is thought to be the direct recognition site for the inositol ring in the product complexes (Fig. 4C)^10^. This residue forms a conserved small side chain (Ala or Ser) in most human enzyme family members, indicating that similar interactions with substrate inositol groups exist. However, the corresponding residue in human INPP5K is Pro152, which forms a bulkier side chain. INPP5K almost exclusively hydrolyses the smaller molecule PtdIns(4,5)P₂, and has much lower turn-over for PtdIns(1,4,5)P₃ and PtdIns(3,4,5)P₃^14,15^. Furthermore, INPP5K does not show significant activity with respect to the soluble inositol phosphates, unlike OCRL and INPP5B. The LC1R and LC2R motifs primarily interact with the aliphatic regions of diC8PtdInsP products. In our crystal structure of Δ INPP5K-cd, the loop corresponding to the LC1R-motif could not be modeled due to a lack of interpretable electron density. This is likely attributable to the high intrinsic flexibility of this region, consistent with the presence of a potential wobble loop. Several amino acid residues in these two motifs differ from those in OCRL and INPP5B, which might affect the substrate preferences of these enzymes.

Knowledge of the structure of human Δ INPP5K-cd will be useful to identify suitable sites for mutational analysis to analyze the relationship between enzyme activity and the degree of severity of CMDs. There is a discrepancy in the findings of CMD studies and those obtained by studying INPP5K knockout mice. It is clear that INPP5K is important for ontogenesis, but its detailed function in adult animals remains to be clarified. The use of conditional knockout mice or chemical biological approach would be expected to provide a better understanding of its roles in adult animals. INPP5K and SHIP2 have recently been reported to act as a driver of cell migration in glioblastoma multiforme^16^, and the studied inhibitor might represent a useful tool for investigating the contribution of INPP5K to cell migration in glioblastoma in vivo^17^.

The structures of IP5Pases are quite similar, and therefore it is difficult to develop selective inhibitors of enzymes in this family through the targeting of substrate binding sites. The studied inhibitor is highly selective for INPP5K, owing to its binding to the allosteric site, which is generated by the repositioning of H3. A hinge exists at the N-terminal of the H3 helix, which involves a hydrophilic amino acid in INPP5K. The inhibitor unexpectedly had no effect on mouse or rat INPP5K. Finally, we identified the most suitable small animal for the evaluation of potential therapeutic substances in vivo, the hamster. This will permit us to investigate the contribution of INPP5K to sarcopenia and glioblastoma multiforme in vivo through the pharmacokinetic optimization of the compounds.

## Methods

### Protein expression and purification for the determination of the crystal structure and HDX analyses of INPP5K

The oligonucleotides used for vector construction were synthesized and purchased from GenScript Inc. (Tokyo, Japan). The cDNA encoding human INPP5K catalytic domain (amino acids 1–328, INPP5K-cd, UniProt ID: Q9BT40), which excluded the SKICH region from the full-length INPP5K sequence, was cloned downstream of MBP (amino acids 1–387) with an N-terminal His6-tag in the pET28 expression vector, a Novagen product (Merck KGaA, Darmstadt, Germany). Between the MBP and INPP5K-cd sequences, the Tobacco Etch Virus protease cleavage site (ENLYFQG) was inserted, such that the MBP–INPP5K-cd fusion protein could be digested by TEV protease during purification. Furthermore, using the Gibson Assembly method, the sequence encoding the amino acids 280–299 (CAGPDTPIPPASHFSLSLRG) of INPP5K cd was replaced by the sequence encoding AKANPSGFLLTQKD, which corresponds to the amino acids 283–296 of the rat INPP5K-cd (UniProt ID: Q9R0B6), resulting in the deletion of six amino acid residues (delta-INPP5K-cd). The truncated construct was designed using a comparison between a structure model and the structures of several IP5Pases^9–11^. The replacement of the sequence was verified using a 3730 DNA Analyzer, an Applied Biosystems product (Thermo Fisher Scientific, Tokyo, Japan). Both proteins were expressed in the E. coli Rosetta2 (DE3) strain, which was cultured in 2 × YT broth at 37 °C to an optical density of ~ 0.6 at 600 nm. Expression was induced by 0.1 mM IPTG for 18 h at 18 °C. The cells were then harvested, resuspended in 50 mL extraction buffer A (100 mM HEPES pH 8.0, 1 mM MgCl2, 500 mM NaCl, 10 mM imidazole, 10% glycerol, 10 mM CHAPS, 0.5 mM TCEP) per liter of cells, and then passed three times through a microfluidizer with the pressure set at 1 000 Pa. The lysate was centrifuged at 42,200 × g for 30 min, and the supernatant obtained was loaded onto a Nickel nitriloacetate (Ni–NTA) Superflow column (Qiagen, Venlo, Netherlands). The column was washed with buffer A, and the bound proteins were eluted using a linear gradient of 0%–100% buffer B (20 mM HEPES pH 8.0, 500 mM NaCl, 500 mM imidazole, 10% glycerol, 10 mM CHAPS, 0.5 mM TCEP). The fractions corresponding to the main peaks were pooled and dialyzed against buffer C (20 mM HEPES pH 7.5, 300 mM NaCl, 2 mM MgCl2, 10% glycerol, 10 mM CHAPS, 0.5 mM TCEP), digested using TEV protease, and then passed through another Ni–NTA column. The INPP5K protein recovered in the flow-through fraction was further purified using a Superdex 200 column (GE Healthcare, Chicago, IL, USA; 16 × 600 mm) in buffer D (20 mM HEPES pH7.5, 300 mM NaCl, 2 mM MgCl2, 10% glycerol, 0.5 mM TCEP). The concentration of protein obtained was measured using the Bradford method, with bovine serum albumin as the standard. Fractions corresponding to each purification step were analyzed by SDS-PAGE, followed by staining of the gel with Coomassie brilliant blue R-250. The purified proteins were concentrated to 1.0 mg/mL, then aliquoted and stored under liquid nitrogen.

We also attempted to prepare two other deletion mutants that corresponded to the sequences of INPP5B and OCRL The amino acids 274–294 (RLKRQPCAGPDTPIPPASHFS) of INPP5K-cd were replaced by KGKN, resulting in the deletion of 17 amino acid residues (INPP5B type delta-INPP5K-cd). In addition, the amino acids 275–294 (LKRQPCAGPDTPIPPASHFS) of INPP5K-cd were replaced by GTN, resulting in the deletion of 17 amino acid residues (OCRL type delta-INPP5K-cd). However, attempts to produce both proteins using the E. coli system resulted in insufficient amounts of the purified proteins for crystallization.

### Hydrogen/deuterium exchange mass spectrometry study of human INPP5K-cd

HDX experiments were performed manually. To initiate the HDX reaction, 2 µl of prechilled protein stock solution at 10 µM was diluted in 18 µl PBS in D2O, and incubated on ice for 15, 50, 150, 500, 1,500, or 5,000 s. At the indicated times, 20 µl of ice-cold acidic buffer (0.8% formic acid) was added to quench the exchange reaction, then the quenched samples were digested with 2.2 µg/mL pepsin for 5 min on ice (mild conditions) to cut out flexible region from INPP5K-cd. The proteolytic products were collected on a C8 trap column for desalting (CAP Trap, 0.5 × 2 mm; Michrom BioResources, Auburn, CA, USA) and eluted on an Easy-Column C18-A1 (20 mm, ID 100 µm, 5 µm; Thermo Fisher Scientific) using a 15-min linear acetonitrile gradient (5%–35%). The eluate was then analyzed using an LTQ mass spectrometer (Thermo Fisher Scientific).

The peptides were identified using Mascot (Matrix Science, Mount Prospect, IL, USA) and the HDX of each peptide was determined using HDExaminer 1.4 (Sierra Analytics Inc., Modesto, CA, USA). In addition, HDX analysis was performed on nondeuterated and fully deuterated samples to correct for back-exchange. The fully deuterated sample was prepared by incubation with deuterated buffer containing 4 M urea at 46.5 °C for 16 h.

### Crystallization and structure determination of human INPP5K

Crystals of the delta-INPP5K-cd/inhibitor complex were obtained at 4 °C in sitting droplets using vapor diffusion against a reservoir containing 0.7–1.0 M malonate pH 5.0–5.4. One mg/mL of purified protein was incubated with a three-times molar excess of inhibitor at 4 °C for 1–2 h, then concentrated to 3 mg/mL for crystallization. Drops consisting of 0.1 µL protein solution and 0.1 µL reservoir solution were equilibrated against 40 µL reservoir solution. Crystals of 20 × 20 × 100 μm^3^ were grown in several successive rounds of micro- and macro-seeding. Unfortunately, we could not obtain apo and complex structures with diC8PtdInsP2 during systematic crystal screening under approximately 10 000 different conditions.

Diffraction data were collected for the delta-INPP5K-cd complexes at BL17A (KEK-PF, Tsukuba, Japan). The crystals were cryoprotected by transfer into 30% ethylene glycol and mounted using a Micro mount loop (MiTe Gen, LLC, Ithaca, NY, USA), frozen in a stream of liquid nitrogen and cooled to 100 K during data collection. The intensity data were integrated with XDS^18^ and scaled using SCALA^19^. The new INPP5K structure was solved using molecular replacement with MOLREP in CCP4 suite using the INPPK5B conformation as the starting model. The structural model was built in Coot^20^ and refined using REFMAC5^21^ in CCP4 suite. The figures were created in PyMOL^22^.

### Protein expression and purification for inhibitor evaluation assays

The protein purification is described in detail elsewhere^3^. Briefly, full-length human INPP5K (amino acids 1–448, UniProt ID: Q9BT40), rat INPP5K (aa 1–446, UniProt ID: Q9R0B6), mouse INPP5K (aa 1–468, UniProt ID: Q8C5L6), hamster INPP5K (aa 1–453, UniProt ID: G3HTF6), guinea pig INPP5K (aa 1–473, UniProt ID: H0V8T7) containing an N-terminal FLAG-tag, full-length human SHIP2 (aa 1–1 258) containing a C-terminal FLAG-tag, and full-length human PIPP (amino acids 1–1 006) containing an N-terminal His8-tag were all cloned into the pcDNA3.4 expression vector and transiently expressed in Expi293 cells (Thermo Fisher Scientific). The PH domain of human TAPP1 (amino acids 180–404) containing an N-terminal GST-tag was expressed in a pGEX6P1 expression vector (GE Healthcare) in E. coli strain BL21 ((Merck KGaA, Darmstadt, Germany)), following IPTG induction at 37 °C for 5 h. All the FLAG-tagged INPP5Ks and hSHIP2 were purified using affinity columns containing DDDDK-tagged protein purification gel (MBL, Medical & Biological Laboratories Co., Ltd., Tokyo, Japan), followed by size-exclusion chromatography with Superdex 200 columns (GE Healthcare). His8-tagged hPIPP was purified using Ni–NTA Superflow (Qiagen) and Superdex 200 columns. The GST-tagged hTAPP1 domain was purified using a Glutathione Sepharose 4B (GE Healthcare) and Superdex 200 columns. The purity of the protein obtained was evaluated using SDS-PAGE.

### Fluorescence polarization assay to evaluate phosphatase inhibition

HTS assays were performed as described previously. Briefly, enzyme reactions were performed in a final volume of 10 µL per well in a 384-well nonbinding black plates (Corning, Corning, NY, USA). The reaction mixtures containing 20 µM dioctanoyl-phosphatidylinositol (3,4,5)-trisphosphate (diC8-PI(3,4,5)P₃) as substrate (Cayman Chemical, Ann Arbor, MI, USA) and each enzyme (human INPP5K, human INPP5K-cd, and human ΔINPP5K-cd) in reaction buffer (50 mM Tris–HCl pH 7.0, 150 mM NaCl, 7.5 mM MgCl₂, 3 mM DTT, 0.03% Triton X-100, 0.03% gelatin), with or without various concentrations of inhibitors, were incubated for 30 min at room temperature. The reactions were stopped by adding 15 µL of detection mix containing 18 mM EDTA, 2.5 nM GloPIPs BODIPY TMR-PI(3,4)P₂ (Echelon Biosciences Inc., Salt Lake City, UT, USA), and 0.1 µM GST–hTAPP1 in detection buffer (PBS containing 0.003% Triton X-100, 0.1% gelatin) and incubating for > 30 min at room temperature. Fluorescence polarization was evaluated using an Envision plate reader (PerkinElmer, U.S. LLC, Shelton, CT, USA) at 531/595 nm (excitation/emission) and calculated as 1 000 × [(I||- (G × I⊥))/(I||+ (G × I⊥))], where I|| is the parallel intensity, I⊥ is the perpendicular intensity, and G = 2.25. The amount of diC8-PI(3,4)P₂ product (P) was determined by standard curve fitting, and the percentage inhibition was calculated using the following equation: 100 × (Pctrl—P)/(Pctrl—Pblank), where Pctrl is the no-inhibitor control and Pblank is the no-enzyme control. Our in-house chemical library, which consists of approximately 360 000 mostly commercially available compounds, was screened using the fluorescence polarization assay system. All IC_50_ values were determined from four independent experiments (n = 4), and data are presented as mean ± SD.

### Inorganic phosphate detection assay for phosphatase inhibition

The release of inorganic phosphate was measured using a PiPer Phosphate Assay Kit (Thermo Fisher Scientific), according to the manufacturer’s instructions, but with some modifications. Enzyme reactions were performed in a final volume of 10 µL per well in a 384-well nonbinding black plates (Greiner Bio-One International, GmbH, Kremsmünster, Austria). The reaction mixtures containing 20 µM diC8-PI(3,4,5)P₃ and each enzyme (human INPP5K, mouse INPP5K, rat INPP5K, hamster INPP5K, guinea pig INPP5K, human SHIP2, or human PIPP) in reaction buffer (50 mM Tris–HCl pH 7.0, 150 mM NaCl, 7.5 mM MgCl₂, 0.03% Igepal CA-630, 0.03% gelatin), with or without various concentrations of inhibitor, were incubated for 20 min at room temperature. The reactions were then stopped and detection reactions were initiated by adding 5 µL of detection mix 1 (60 mM EDTA, 12 U/mL maltose phosphorylase, and 0.6 mM maltose in 100 mM Tris–HCl pH 8.0). After incubation for 60 min at room temperature, 5 µL of detection mix 2 (8 U/mL glucose oxidase, 0.8 U/mL HRP, and 100 µM Amplex Red in 100 mM Tris–HCl pH 8.0) was added, and the mixture was incubated for a further 30 min at room temperature in the dark. The fluorescence generated was measured using an Envision plate reader at 531/590 nm (excitation/emission), and the percentage inhibition was calculated as described above.

## Supplementary Information

Supplementary Information.

## Data Availability

The atomic coordinates and structure factors for the crystal structure reported in this paper have been deposited in the Protein Data Bank (PDB) under accession code 22 MJ. These data will be released upon publication. All other data supporting the findings of this study are available from the corresponding author upon reasonable request.
