# Supplementary material for: Crystal structure of human INPP5K with an allosteric inhibitor reveals the structural basis for species specific potency
Source: Sci Rep. 2026 Feb 26;16:11132. doi: 10.1038/s41598-026-40748-4 (PMC13047053; doi:10.1038/s41598-026-40748-4)
Supplement: Supplementary file 1 — Supplementary Information. [file 41598_2026_40748_MOESM1_ESM.docx]

**Supplementary material**

**Crystal structure of human INPP5K with an allosteric inhibitor reveals the structural basis for species specific potency**

Akihiro Nomura, Keishi Yamaguchi, Motoaki Kawano, Kazuki Hanada, Jun Nishihata, Masato Noguchi, and Tsuyoshi Adachi

**Methods**

***Computational modeling and structure prediction***

*Structural modeling for construct design*

To guide the design of a crystallizable construct, the three-dimensional structure of the human INPP5K catalytic domain (INPP5K-cd, residues 1–328) was modeled using the Boltz-2 algorithm [11]. The modeling was performed using the default parameters, without an explicit template. The resulting model predicted a large, solvent-exposed loop region (residues 276–293) with a low confidence score, suggesting a high level of flexibility. This region was subsequently targeted for replacement with a shorter sequence from rat INPP5K to create the ΔINPP5K-cd construct, aiming to increase its propensity to crystallize.

*Structure prediction of the INPP5K–CPD-1 complex*

To assess whether computational methods could predict the site of the novel allosteric pocket, structure prediction of the complex of CPD-1 and the apo structure of human ΔINPP5K-cd (as determined in the present study, with CPD-1 and malonate removed) was performed using Boltz-2. The entire protein surface was treated as the searchable space to allow for unbiased identification of potential binding sites. A total of 10 distinct models were generated and ranked using the model’s predicted affinity score. None of the top-10 scoring models predicted the experimentally identified allosteric site, which was located at the interface of the β-sheet core and helix H3. This highlights the challenge for current algorithms associated with the prediction of binding events that involve significant induced-fit conformational changes, as illustrated in Supplementary Figure 5.

*Overall structures of human ΔINPP5K-cd and INPP5B*

Define Secondary Structure of Proteins (DSSP) analysis revealed the following principal secondary structure elements for human ΔINPP5K-cd (Supplementary Figure 1): S1 (Lys5–Leu6), S2 (Arg12–Asn22), H1 (Ser33–Leu36), S3 (Ile47–Gln53), H2 (Ser71–Leu80), S4 (Phe85–Met93), S5 (Ile96–Lys103), S6 (Ile110–Pro119), S7 (Gly130–Leu138), S8 (Tyr141–His149), H3 (Leu161–Gln171), S9 (Leu186–Asp192), H4 (Leu202–Lys210), H5 (Gly215–Lys220), H6 (Gln222–His229), S10 (Asp269–Leu275), S11 (Phe287–Ser296), S12 (Val308–Leu316), and S13 (Leu319–Val320). The compound was located between H3 and the β-sheet core domain. The loop between S10 and S11 was found to vary in length between species and other IP5Pases. The amino acid residues 280–299 (CAGPDTPIPPASHFSLSLRG) were replaced by AKANPSGFLLTQKD, resulting in a construct which six amino acid residues had been deleted.

DSSP analysis revealed the following principal secondary structure elements for INPP5B (PDB code: 4CML) (Supplementary Figure 2): S1 (Thr260-Asn273), H1′ (Arg284–Leu287), S2 (Val296–Gln302), H1 (Lys308–Phe311), H2 (Pro317–Gly329), S3 (Tyr336–Leu344), S4 (Ile347–Lys354), S5 (Ile361–Gly370), S6 (Gly381–Phe389), S7 (Thr392–His400), H3 (Tyr408–Arg421), S8 (Gln423–Phe424), S9 (Leu434–Thr435), S10 (Val441–Asp447), H4 (Val457–Glu465), H5 (Phe469–Tyr473), H6 (Gln477–Ala483), S11 (Asp524–Gly530), S12 (Ile533–Ser540), and S13 (Val552–Val563). The loop between S11 and S12 is shorter than that of INPP5K (Supplementary Figure 2).

***Chemical characterization of CPD-1***

^1^H-NMR spectra were recorded on a JEOL Resonance ASC100 and ^13^C-NMR spectra were recorded on a Bruker Avance Neo 500 at 25°C. HRMS spectra were recorded on an LC/MS system composed of an Agilent 1290 and a Thermo Fisher Orbitrap ID-X (EAA26).

2-Benzamido-5-isopropyl-4-phenylthiophene-3-carboxylic acid (CPD-1), ^1^H-NMR (400 MHz, DMSO-*d_6_*) δ: 12.84 (1H, br s), 12.45 (1H, br s), 7.93 (2H, dd, *J* = 6.7, 1.9 Hz), 7.71–7.69 (1H, m), 7.65–7.62 (2H, m), 7.40–7.38 (2H, m), 7.34–7.31 (1H, m), 7.21 (2H, dd, *J* = 6.5, 1.6 Hz), 2.92–2.82 (1H, m), 1.16 (6H, d, *J* = 7.0 Hz); ^13^C-NMR (125 MHz, DMSO-*d_6_*) δ 167.1, 162.8, 145.7, 138.8, 136.9, 133.7, 132.7, 132.1, 129.4, 129.2, 127.5, 126.9, 126.7, 111.3, 27.3, and 24.7. The HRMS calculated for C_21_H_19_NO_3_S [M-H]^+^ was 364.1013, and that measured was 364.1014.

**Supplementary Table 1. Percent identity matrix of INPP5K catalytic domains across species**
The sequence identity percentages were calculated using Clustal 2.1

| **Species** | **Human** | **Hamster** | **Guinea pig** | **Mouse** | **Rat** |
| --- | --- | --- | --- | --- | --- |
| **Human** | 100.00 |  |  |  |  |
| **Hamster** | 75.06 | 100.00 |  |  |  |
| **Guinea pig** | 70.64 | 72.05 | 100.00 |  |  |
| **Mouse** | 75.67 | **87.36** | 71.27 | 100.00 |  |
| **Rat** | 75.74 | **86.46** | 71.66 | 90.81 | 100.00 |

**Note:**
Values indicate the percentage of identical residues between the sequences.
**Bold values** indicate the high sequence identity between Hamster and Mouse/Rat orthologs (>86%), supporting the "natural mutagenesis" comparison discussed in the main text. Specifically, Hamster INPP5K shares significantly higher identity with Mouse/Rat enzymes than with the Human enzyme (approx. 75%), yet exhibits high inhibitor sensitivity similar to the Human enzyme.

**Supplementary Table 2. Comparison of residues constituting the inhibitor-binding pocket**
Detailed comparison of residues located within 3.7 Å of CPD-1 in the crystal structure and the corresponding residues in other species.

| **Residue (Human No.)** | **Human** | **Hamster (Sensitive)** | **Guinea pig (Low sens.)** | **Mouse (Insensitive)** | **Rat (Insensitive)** | **Conservation** |
| --- | --- | --- | --- | --- | --- | --- |
| **Thr 118** | **Thr** | **Ile** | **Thr** | **Thr** | **Ile** | **Varied^a^** |
| **Gly 130** | **Gly** | **Gly** | **Gly** | **Gly** | **Gly** | **Conserved** |
| **Gly 131** | **Gly** | **Gly** | **Gly** | **Gly** | **Gly** | **Conserved** |
| **Leu 150** | **Leu** | **Leu** | **Leu** | **Leu** | **Leu** | **Conserved** |
| **Arg 160** | **Arg** | **Arg** | **Arg** | **Arg** | **Arg** | **Conserved** |
| **Phe 164** | **Phe** | **Phe** | **Phe** | **Phe** | **Phe** | **Conserved** |
| **Ile 167** | **Ile** | **Ile** | **Ile** | **Ile** | **Ile** | **Conserved** |
| **Gln 171** | **Gln** | **Gln** | **Gln** | **Leu** | **Leu** | **Different** |
| **Glu 175** | **Glu** | **Glu** | **Lys** | **Glu** | **Glu** | **Different** |
| **Trp 189** | **Trp** | **Trp** | **Trp** | **Trp** | **Trp** | **Conserved** |
| **Ala 226** | **Ala** | **Ala** | **Ala** | **Ala** | **Ala** | **Conserved** |
| **Leu 233** | **Leu** | **Leu** | **Leu** | **Leu** | **Leu** | **Conserved** |

**Notes:**
**a)** The variation at position 118 (Thr vs. Ile) does not correlate with inhibitor sensitivity. For example, the mouse enzyme is insensitive despite having Thr (same as human), whereas the hamster enzyme is sensitive despite having Ile.

Supplementary Figures


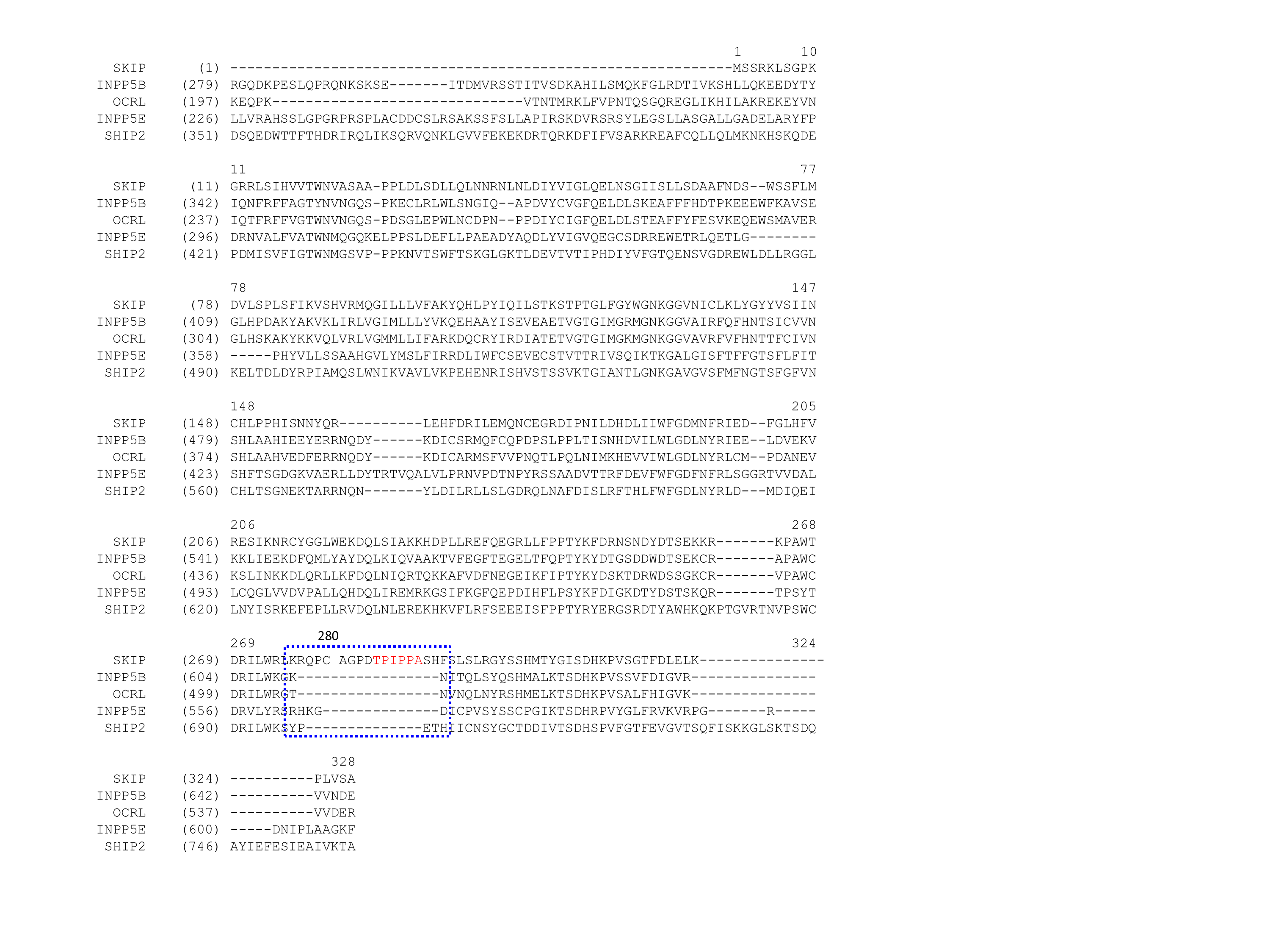


**Supplementary Figure 1. Sequence alignment of human IP5Pase catalytic domains**

Alignment of the catalytic domains of human INPP5K (residues 1–328), INPP5B, OCRL1, SHIP2, and INPP5E. The predicted flexible loop region that is unique to INPP5K is indicated by the blue dotted square box.


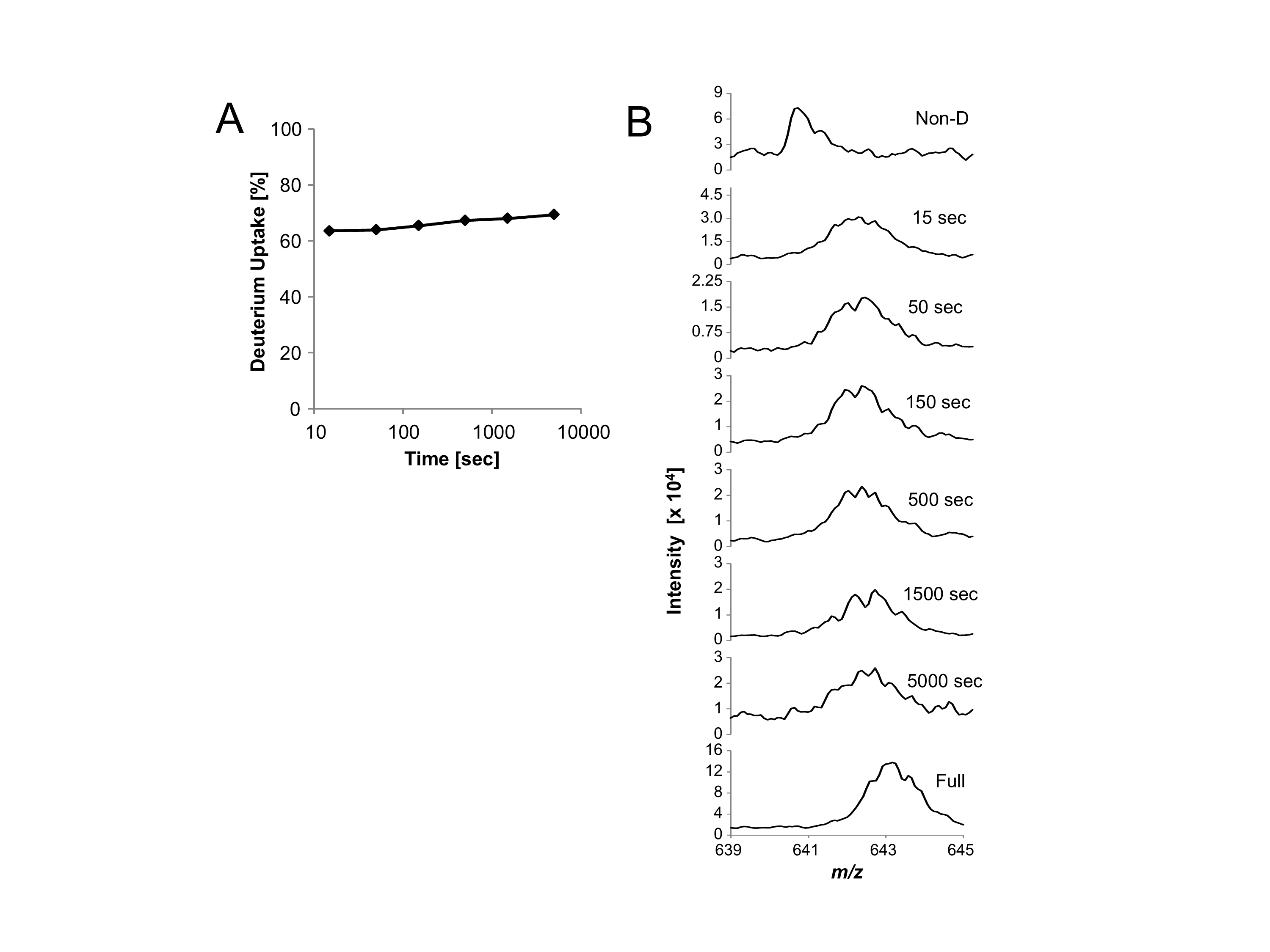


**Supplementary Figure 2. HDX–MS analysis of the predicted flexible loop region in human INPP5K-cd**

(A) Deuterium uptake curve for a representative peptide fragment covering the predicted flexible loop (residues 276–293). This fragment shows relatively high and rapid deuterium exchange, characteristic of a disordered region, when compared with peptides derived from stable secondary structure elements, such as α-helices and β-sheets, within human INPP5K-cd. (B) Representative mass spectra of the selected peptide at increasing deuterium exchange times (indicated). Deuterium uptake shifts the isotopic envelope to higher mass-to-charge (m/z) values in a time-dependent manner. The deuterium uptake percentage at each time point was calculated relative to nondeuterated (defined as 0% uptake) and fully deuterated (defined as 100% uptake) controls.


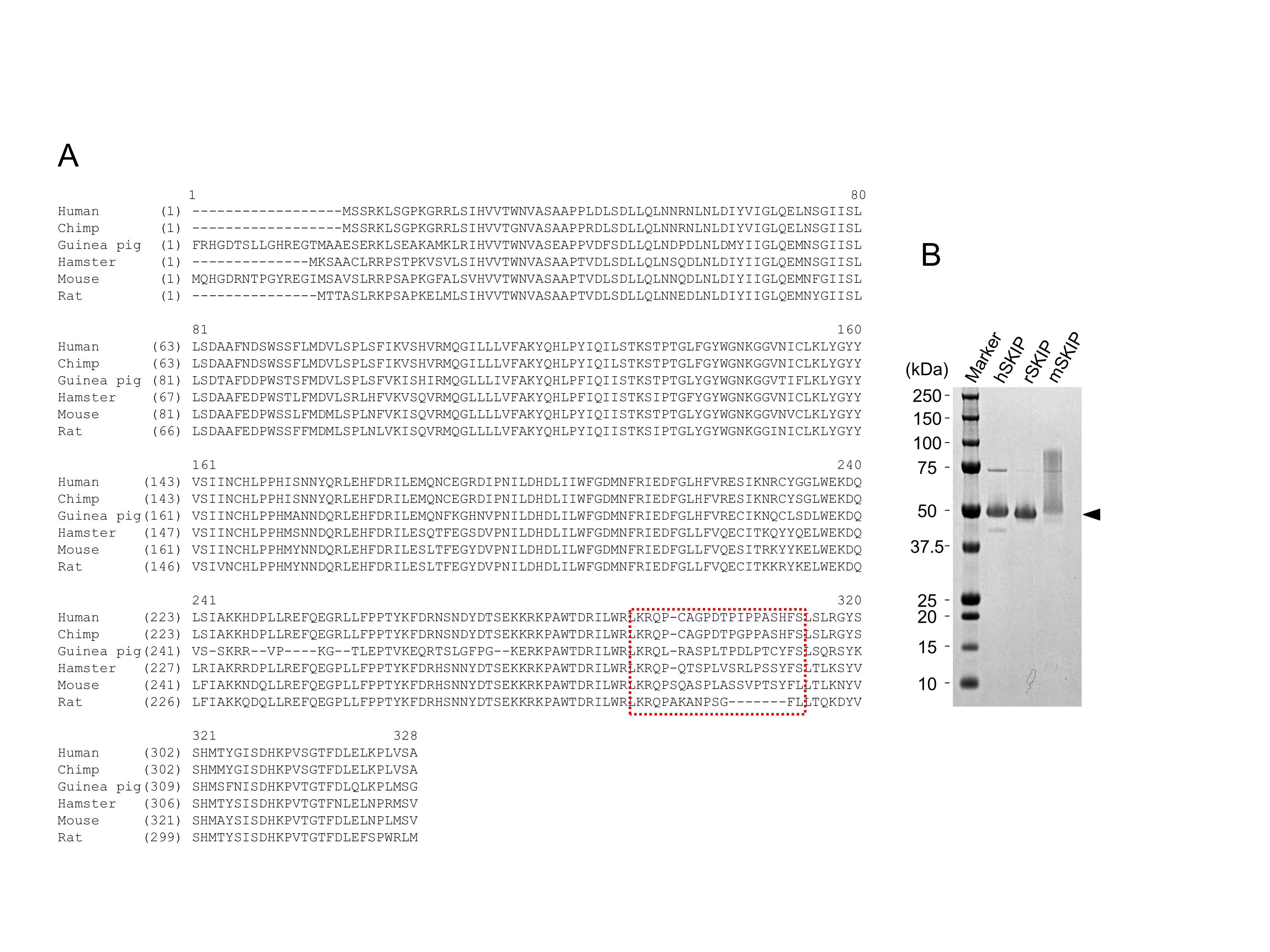


**Supplementary Figure 3. Sequence alignment and purification analysis of INPP5K orthologs**

(A) Sequence alignment of the catalytic domains of human, mouse, and rat INPP5K. Sequence variation among the orthologs, particularly within the predicted flexible loop region (indicated by the red dotted line), is highlighted. (B) SDS–PAGE analysis of the purified INPP5K catalytic domain constructs. The mouse INPP5K protein exhibits apparent variation in molecular weight, in contrast to the human and rat proteins, under these conditions.


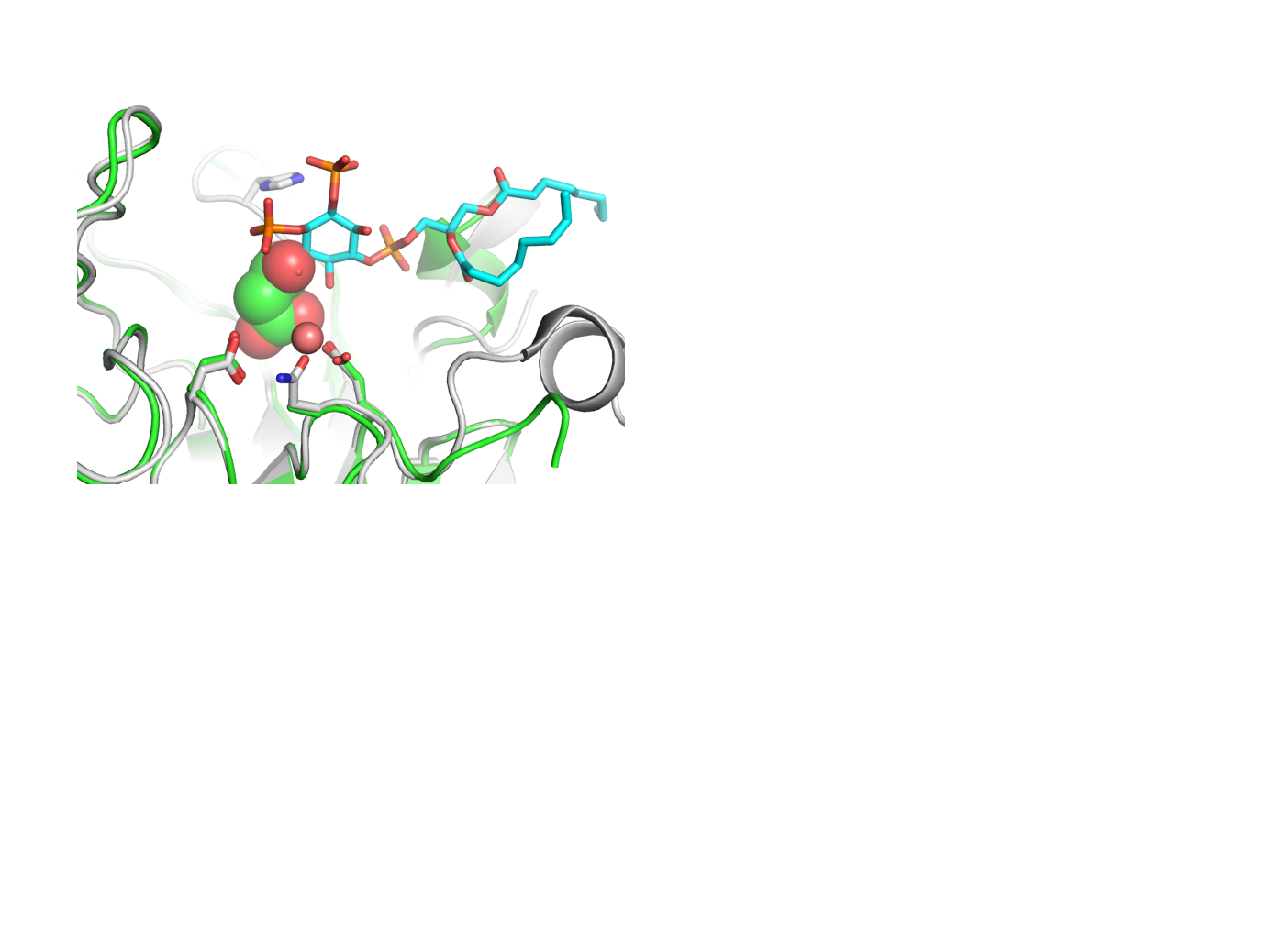


**Supplementary Figure 4. Malonate occupancy in the active site of ΔINPP5K-cd**

Superimposition of the INPP5B–diC8PtdInsP_2_ complex (PDB: 4CML, protein shown as white ribbon; product of INPP5B–diC8PtdInsP_2_, shown as cyan sticks) onto the human ΔINPP5K-cd structure (green ribbon). Malonate (green and red spheres), which was present in the crystallization buffer, was bound in the active site cleft of ΔINPP5K-cd. Its position likely causes steric hindrance, potentially interfering with the binding of substrates or products, such as INPP5B–diC8PtdInsP_2_.


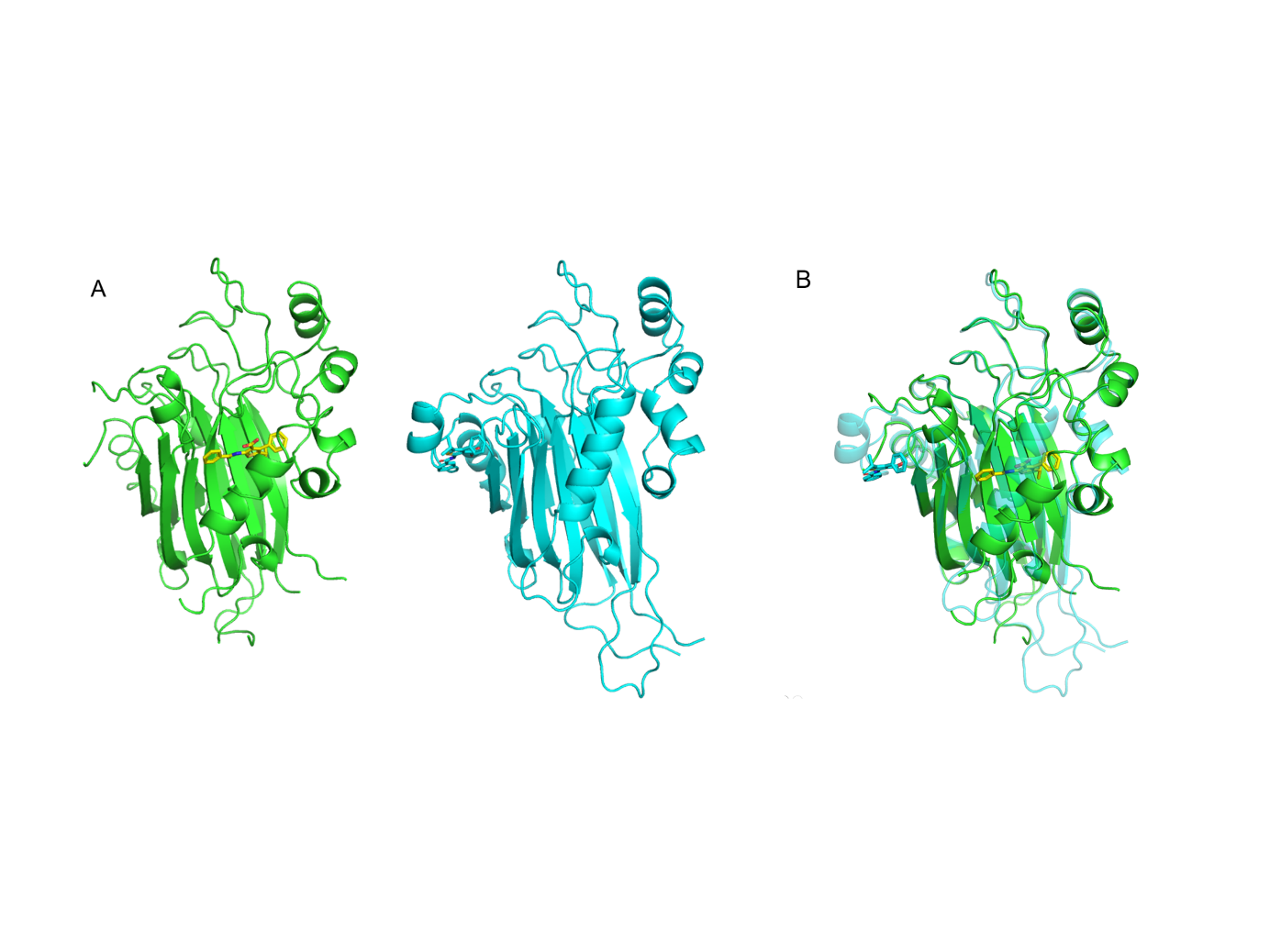


**Supplementary Figure 5. Comparison of the computationally predicted binding pose of CPD-1 with its crystal structure**

(A) Side-by-side comparison of the experimentally determined crystal structure of the complex with CPD-1 (green) and a representative binding model predicted by the Boltz-2 docking simulation (cyan). (B) Superimposition of the predicted model onto the crystal structure. The overlay clearly demonstrates that the predicted binding location and orientation are completely different from the pose identified in the allosteric pocket. Notably, this stark discrepancy was not limited to a single pose; all of the top 10 scoring poses generated by Boltz-2 similarly failed to identify the correct binding site.

***
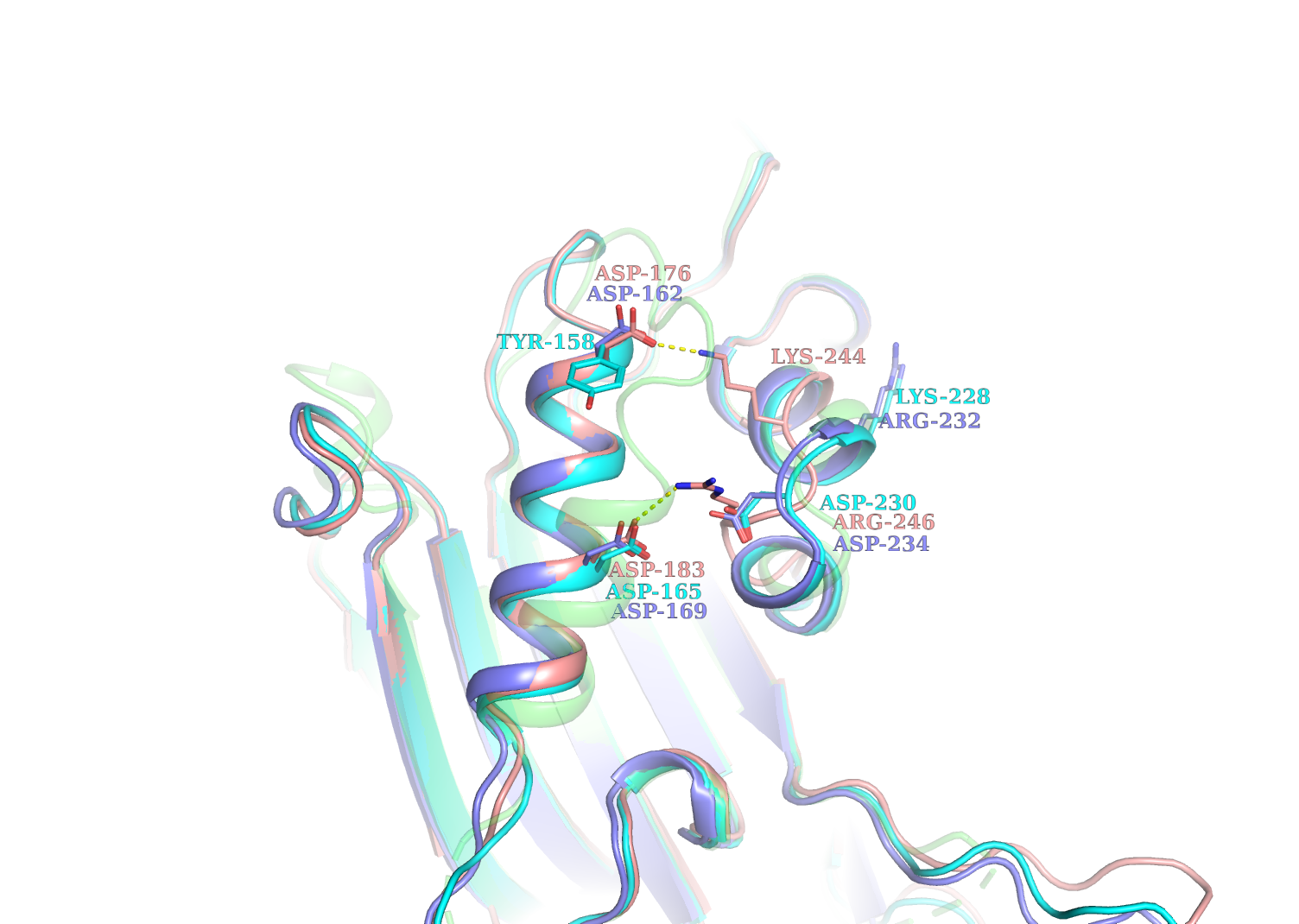
***

**Supplementary Figure S6. Structural basis for the "electrostatic brake" in guinea pig INPP5K**

Superimposition of the AlphaFold 3 predicted closed conformations of guinea pig (pink), human (cyan), and hamster (slate) INPP5K catalytic domains. The crystal structure of the human INPP5K–CPD-1 complex (open conformation) is shown in semi-transparent green.

In the guinea pig structure, basic residues Lys244 and Arg246 form salt bridges (yellow dashed lines) with aspartates (Asp176 and Asp183) on helix H3. This "electrostatic brake" stabilizes the closed conformation.

In contrast, in both human and hamster structures, the residue corresponding to the braking Arg246 is replaced by an aspartate (Asp230 in human, Asp234 in hamster). This substitution eliminates the stabilizing salt bridge observed in the guinea pig. Furthermore, the proximity of these aspartates to acidic residues on helix H3 (Asp165 in human, Asp169 in hamster) likely generates electrostatic repulsion, destabilizing the closed state and facilitating the conformational change required for inhibitor binding.
